# Complex intraoperative defect planning flexibility—Post-salvage laryngectomy defect reconstruction with a double skin-paddled radial forearm free flap

**DOI:** 10.1016/j.jpra.2024.06.004

**Published:** 2024-06-12

**Authors:** Wiktor Teodor Pilch, Nandakumaran Kandamany

**Affiliations:** Royal Hobart Hospital, Department of Plastic and Reconstructive Surgery, Hobart, Tasmania, 7000 Australia

**Keywords:** Salvage laryngectomy, Reconstruction, Radial forearm free flap, Double skin-paddled free flap, Head and neck surgery, Microsurgery

## Abstract

Reconstruction of pharyngoesophageal defects following total laryngectomy poses a significant challenge. The goals of reconstruction are to provide alimentary tract continuity and to restore speech and swallowing functions. Patients with radiotherapy recurrent disease often have unfavourable tissue for healing with a high incidence of pharyngocutaneous fistula. We discuss utilisation of a double skin paddle radial forearm free flap for pharyngoesophageal reconstruction as well as a cutaneous skin defect.

A 53-year-old female was referred to our department for reconstruction of her total laryngectomy defect secondary to radio-recurrent right laryngeal squamous cell carcinoma with extra-laryngeal spread.

Reconstruction planning was challenging as the patient was an obese, heavy smoker with significantly irradiated neck skin. A tubed radial artery forearm free flap was planned for pharyngoesophageal reconstruction however due to the extent of radiotherapy skin damage; primary closure of the neck defect was not possible. The flap was modified into a double paddle design to reconstruct the pharyngoesophageal defect, with the second skin paddle folded over to reconstruct the cutaneous defect.

Multiple reconstructive options have been described in the literature for primary laryngectomy defects. Complex patients with recurrence particularly after neoadjuvant treatment are often poor candidates for reconstruction with poor tissue viability. Providing sufficient and adequate soft-tissue coverage is essential to minimise complications. We have described an intra-operatively planned, novel technique of reconstruction. Pre-operative anticipation may assist in addressing complexities encountered particularly in settings of hostile native skin.

Reconstruction of pharyngoesophageal defects following total laryngectomy poses a significant challenge. The goals of reconstruction are to provide alimentary tract continuity and to restore speech and swallowing functions.^1^ Patients with radiotherapy recurrent disease often have unfavourable tissue for healing with a high incidence of pharyngocutaneous fistula.^2^ We discuss an on-table designed double skin paddle radial forearm free flap (RAFF) for pharyngoesophageal reconstruction, and cutaneous skin defect reconstruction.

## Case Report

A 53-year-old female was referred to our department for reconstruction of her total laryngectomy defect secondary to radio-recurrent right laryngeal squamous cell carcinoma with extra-laryngeal spread. This is on the background of a locally advanced right vocal cord p16 negative SCC following a history of otalgia and dysphonia. She completed a course of chemoradiotherapy (cisplatin) with subsequent significant local disease progression including subglottic, glottic and supraglottic lymph nodes requiring a tracheostomy.

Reconstruction planning was challenging as the patient was a morbidly obese (140 kg), heavy smoker (37 pack year history) with hostile irradiated neck skin.

**Figure 1:**
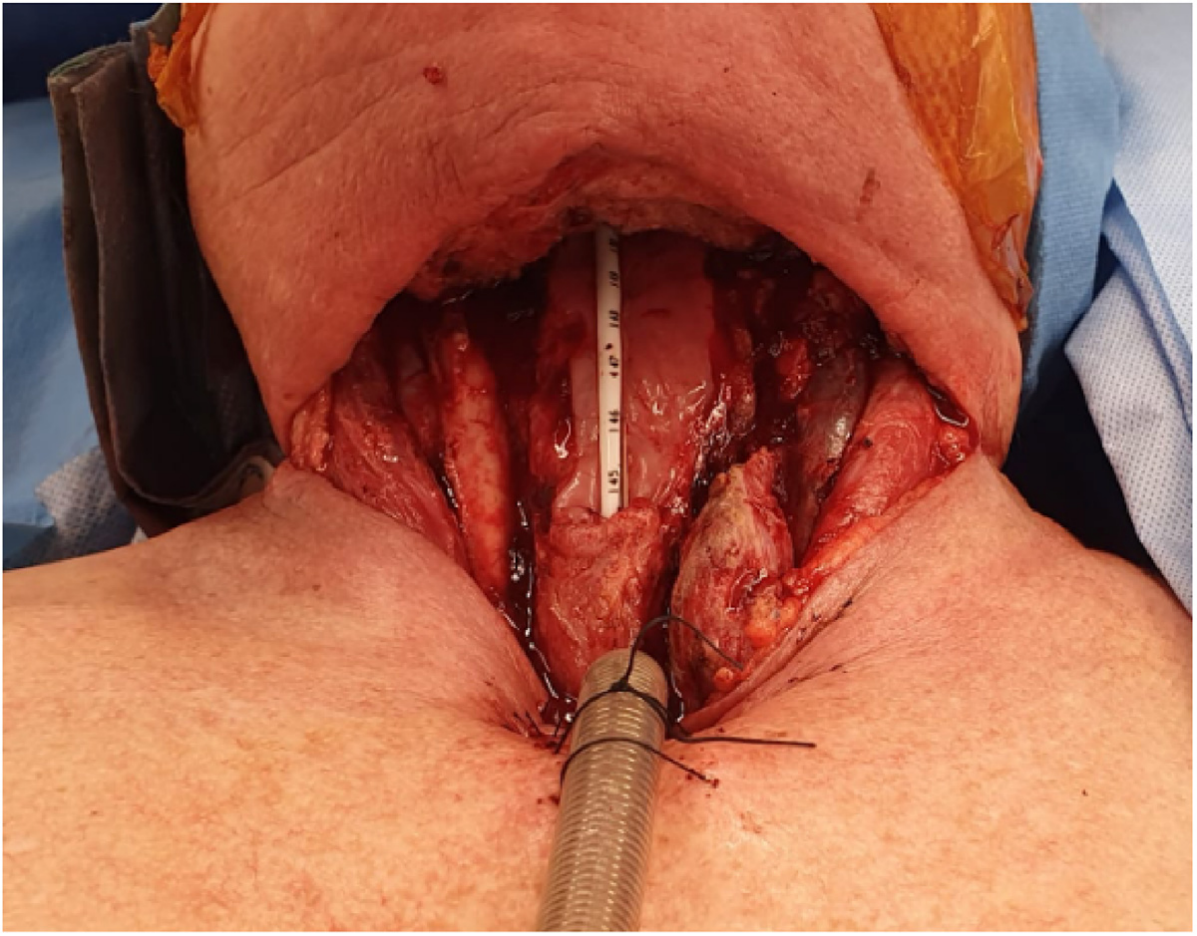
Anterior defect post- laryngectomy requiring reconstruction.

The patient underwent a panendoscopy, total laryngectomy, bilateral level 2–4 selective neck dissections, right hemithyroidectomy, primary tracheooesophageal puncture and cricomyotomy with the Otolaryngology team leaving a significant anterior defect (Figure 1).

Three centimetres of posterior oesophageal wall remained, with a small base of tongue defect as well as a significant anterior neck cutaneous defect from oedematous and previously irradiated tissues. Reconstruction would require closure of the pharyngooesophageal defect initially followed by closure of the cutaneous defect. A standard tubed radial artery forearm free flap (RAFF) was planned for pharyngoesophageal reconstruction however due to the extent of radiotherapy skin damage; primary closure of the neck defect was not possible. Options included consideration of utilisation of a single large anterolateral thigh free flap, two combined free flaps, or a free flap beneath a pedicled pectoralis major flap with cutaneous defect coverage. Only the tubed radial forearm free flap was considered thin and size appropriate to reconstruct her pharyngooesophageal defect, secondary coverage with a subsequent free or local flap was considered a significantly increased morbidity.

On table the decision was made to attempt to modify the flap into a double paddle design to reconstruct the pharyngoesophageal defect, with the second skin paddle folded over to reconstruct the cutaneous defect. This was the preferable donor site given the patient's body habitus, and skin availability.

**Figure 2:**
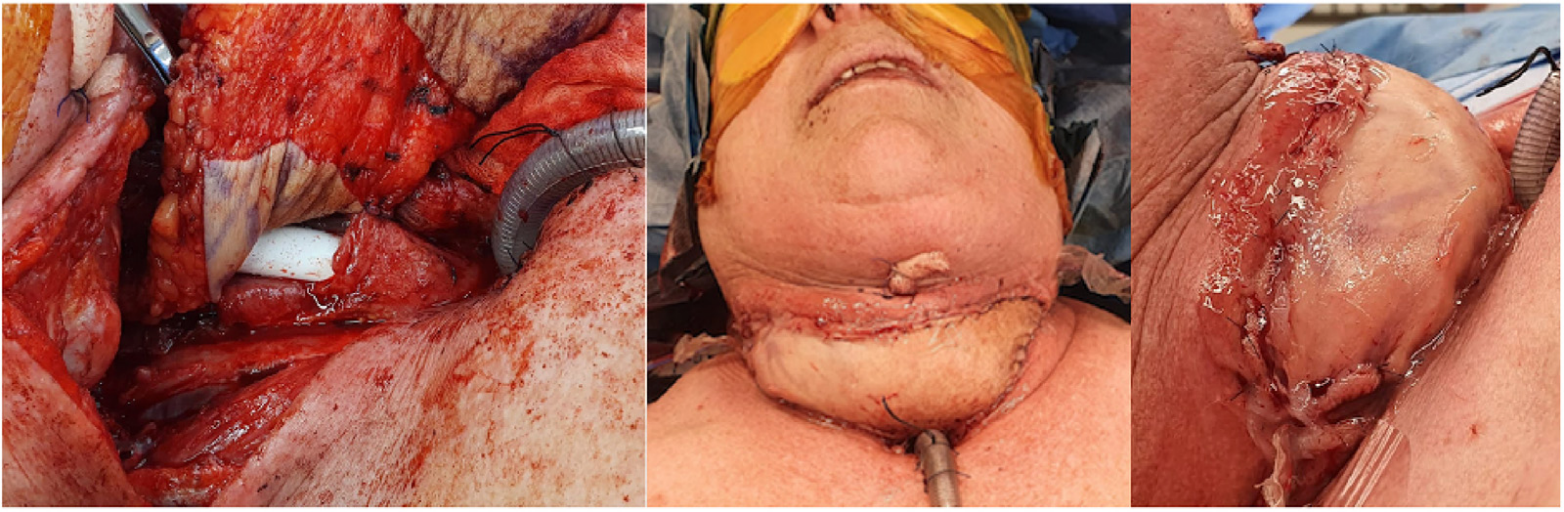
Partially de-epithelialised RAFF to create two skin paddles; the tubed skin paddle was inset to recreate the neooesophagus, with the second skin paddled folded over to fill the skin defect.

RAFF was partially de-epithelialised to create two skin paddles; the tubed skin paddle was inset to recreate the neooesophagus, with the second skin paddled folded over to fill the skin defect (Figure 2). Anastomoses included the facial artery, cephalic vein and retromandibular veins. A Montgomery salivary bypass tube was inserted and secured.

This provided robust, non-irradiated skin for both monitoring and sealed wound closure. Defect size was 17 cm x 21 cm, which was covered with BTM (Polynovo) and skin grafted after delamination at week 6, at the same time as the planned removal of the Montgomery salivary bypass tube in the operating theatres under anaesthetic.

**Figure 3:**
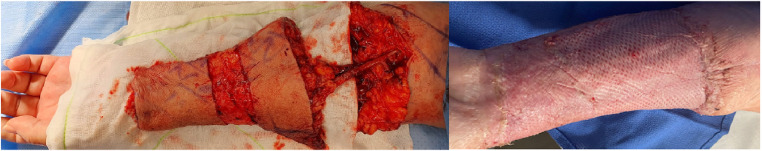
In-situ double paddle design for a tubed RAFF, as well as the defect 2 weeks post BTM delamination and skin grafting- with complete take and robust skin.

Figure 3 shows the in-situ double paddle design for a tubed radial artery forearm free flap as well as the defect 2 weeks post BTM delamination and skin grafting- with complete take and robust skin.

## Discussion

Multiple reconstructive options have been described in the literature for primary laryngectomy defects.^3^ Complex patients with recurrence particularly after neoadjuvant treatment are often poor candidates for reconstruction with poor tissue viability. Providing sufficient and adequate soft-tissue coverage is essential to minimise complications. Pre-operative anticipation as well as intra-operative flexibility may assist in addressing the complexities encountered, particularly in the setting of hostile native skin.

## Declaration of competing interest

None.
